# Treatment of diabetic retinopathy through neuropeptide Y‐mediated enhancement of neurovascular microenvironment

**DOI:** 10.1111/jcmm.15016

**Published:** 2020-03-06

**Authors:** Kepeng Ou, David A. Copland, Sofia Theodoropoulou, Sonja Mertsch, Youjian Li, Jian Liu, Stefan Schrader, Lei Liu, Andrew D. Dick

**Affiliations:** ^1^ College of Pharmacy National and Local Joint Engineering Research Center of Targeted and Innovative Therapeutics Chongqing Key Laboratory of Kinase Modulators as Innovative Medicine Chongqing University of Arts and Sciences Chongqing China; ^2^ Laboratory for Experimental Ophthalmology University of Düsseldorf Düsseldorf Germany; ^3^ Academic Unit of Ophthalmology Bristol Medical School University of Bristol Bristol UK; ^4^ Department of Ophthalmology Faculty of Medicine and Health Sciences University of Oldenburg Oldenburg Germany; ^5^ National Institute for Health Research (NIHR) Biomedical Research Centre at Moorfields Eye Hospital University College London Institute of Ophthalmology London UK

**Keywords:** diabetic retinopathy, neurovascular unit, NPY, retinal ganglion cells, vascular permeability, ZO‐1

## Abstract

Diabetic retinopathy (DR) is one of the most severe clinical manifestations of diabetes mellitus and a major cause of blindness. DR is principally a microvascular disease, although the pathogenesis also involves metabolic reactive intermediates which induce neuronal and glial activation resulting in disruption of the neurovascular unit and regulation of the microvasculature. However, the impact of neural/glial activation in DR remains controversial, notwithstanding our understanding as to when neural/glial activation occurs in the course of disease. The objective of this study was to determine a potential protective role of neuropeptide Y (NPY) using an established model of DR permissive to N‐methyl‐D‐aspartate (NMDA)‐induced excitotoxic apoptosis of retinal ganglion cells (RGC) and vascular endothelial growth factor (VEGF)‐induced vascular leakage. In vitro evaluation using primary retinal endothelial cells demonstrates that NPY promotes vascular integrity, demonstrated by maintained tight junction protein expression and reduced permeability in response to VEGF treatment. Furthermore, ex vivo assessment of retinal tissue explants shows that NPY can protect RGC from excitotoxic‐induced apoptosis. In vivo clinical imaging and ex vivo tissue analysis in the diabetic model permitted assessment of NPY treatment in relation to neural and endothelial changes. The neuroprotective effects of NPY were confirmed by attenuating NMDA‐induced retinal neural apoptosis and able to maintain inner retinal vascular integrity. These findings could have important clinical implications and offer novel therapeutic approaches for the treatment in the early stages of DR.

## 1 INTRODUCTION

Diabetic retinopathy (DR) is the leading cause of visual loss in adults aged 20‐74 years.^1^ The incidence of DR is expected to rise further due to the increasing prevalence of diabetes, ageing of the population and increase in the life expectancy of individuals with diabetes.^2^ Conventional clinical assessment and classification is based on classical microvascular features including the following: haemorrhage, lipid exudate, cotton wool spots and neovascularization, all observed predominantly in the inner retina.^3^ However, pre‐clinical studies also demonstrate that components of the neurovascular unit, including the inner and outer neurosensory retina, are disrupted in diabetes.^4^, ^5^ Perturbed neuronal function reflected by impaired glutamatergic and dopaminergic neurotransmitter signalling,^6^ altered dendritic fields^7^ and reduced synaptic protein expression has been documented.^8^ These changes ultimately leading to apoptosis of neurons alongside persistent uncontrolled diabetes.^9^ Additional diabetic changes include altered glial cell activation, demonstrated by impaired interconversion of glutamate and glutamine,^10^ regulation of potassium channels^11^ and subsequent expression of the glutamate‐aspartate transporter and intermediary filament proteins such as glial fibrillary acidic protein (GFAP). Diabetes also induces changes to retinal astrocytes, which located in the retinal nerve fibre layer and aligned with blood vessels provide contact with synapses,^12^ and where connexin expression is reduced in the early course of diabetes prior to astrocyte loss.^13^ Collectively, diabetes causes components of the retinal neurovascular unit to ‘dis‐integrate’, and accordingly, DR should be recognized as a neurovascular degeneration and not solely a microvascular disease.^14^, ^15^ Nevertheless, recognition that disruption of the neurovascular unit presents opportunities for new therapeutic strategies and/or molecular targets that may offer potential for treating in the early stages of disease.

Neuropeptide Y (NPY) is one of the most abundant peptides in the mammalian central nervous system (CNS).^16^ NPY is a highly conserved 36 amino acid peptide which binds to a family of G protein–coupled receptors Y1‐6,^17^ expressed in the retina of several species including mice.^18^, ^19^, ^20^, ^21^ Studies demonstrate putative neuroprotective effects of NPY in different CNS regions,^22^ including inhibition of glutamate release in rat hippocampus and striatum.^23^ Selective activation of NPY receptors has also been shown to protect mouse hippocampal cells from excitotoxic lesions.^24^ Specific activation of Y2 receptor is effective in a transient middle cerebral artery occlusion model of ischaemia,^25^ and NPY receptor signalling suppresses glutamate‐induced necrosis and apoptosis in retinal neural cells,^26^ modulates retinal ganglion cell (RGC) physiology and elicits neuroprotective effects in vitro*.* 27 Although no direct evidence in retinal endothelium, NPY can induce migration and proliferation of endothelial cells via its receptors and regulate their function.^28^ Conversely, a recent study demonstrates that NPY treatment in a model of acute retinal ischaemia reduced retinal function and tissue damage.^29^

In light of the reported decrease in mRNA and protein levels for NPY in diabetic retina,^30^ as well as observations of NPY function, we examined whether administration of NPY would extend and offer protection of the neurovascular multicellular complex in the diabetic retina. Using in vitro platforms, we first assessed whether neuroprotective actions of NPY could reduce the sensitivity of retinal neurons to glutamate‐induced excitotoxicity. Additionally, we evaluated the potential intracellular signalling pathways of NPY suppression of vascular endothelial growth factor (VEGF)‐induced vascular permeability in retinal endothelial cells. Finally, we established an in vivo model of DR, permissive to accelerated glutamate excitotoxicity and VEGF‐induced vascular leakage to evaluate whether NPY is neuroprotective, attenuating retinal neural apoptosis and able to maintain inner retinal vascular integrity.

## 2 MATERIALS AND METHODS

### 2.1 Cell culture and viability

Primary Retinal Microvascular Endothelial cells from mouse (MRMECs) or human (HRMECs) were purchased from company (Generon) and were cultured in Complete Mouse and Human Endothelial Cell Medium, respectively (Generon). We used the MTT assay to assess cell viability.

### 2.2 In vitro vascular permeability assay

Permeability visualization experiments were performed on 18 × 18 mm glass coverslips as previously described.^31^ For assays, serum‐free culture medium supplemented with the treatment (combinations of NPY, N‐methyl‐D‐aspartate [NMDA], VEGF) was added for a further 24 hours. Then, fluorescein‐streptavidin was directly added to the culture medium for 5 minutes and washed twice with PBS before cell fixation with 3.7% paraformaldehyde (PFA) in room temperature (RT) subjected to immunofluorescence staining according to the manufacturer's instructions.

### 2.3 SDS‐PAGE analysis

For analysis of protein levels, cultured MRMECs cells were rinsed twice with ice‐cold PBS before lysis with ice‐cold CelLytic™ MT Cell Lysis Reagent (Sigma‐Aldrich). Cell lysates were centrifuged at 14 000 *g* for 10 minute at 4°C, the supernatant was transferred to fresh tubes, and Pierce BCA Protein Assay Kit (Thermo Fisher Scientific) was used to determine the protein concentration in each sample. Then, the equal protein was mixed with 4× SDS sample buffer, boiled for 10 minutes at 90°C and resolved using 4% to 20% NuPAGE gels (Invitrogen). Proteins were transferred to PVDF membranes (Invitrogen), blocked with 5% skimmed milk for 2 hours, rinsed and incubated overnight at 4°C with the following primary antibodies: anti‐ZO‐1 (ab2272, Merck, UK; 1:1000), anti‐p38 MAPK (#8690, Cell Signaling Technology; 1:1000), anti‐p44/42 MAPK (#4695, Cell Signaling Technology; 1:1000) and β‐actin (Cell Signaling Technology; 1:1000). Excess antibody was then removed by washing the membrane in PBS/ 0.1% Tween 20, and the membranes were incubated for 2 hours with horseradish peroxidase‐conjugated secondary antibodies (Abcam, UK; 1:2000). Following further washes in PBS/0.1% Tween 20, protein signals were developed with ECL reagent (Sigma) and captured by an electronic imaging system (Konica Minolta). Restore™ PLUS Western Blot Stripping Buffer (Thermo Fisher Scientific) was used to re‐blot the membranes. The density of protein was quantified using the ImageJ.

### 2.4 Retinal explant culture ex vivo model

Retinal explant culture was performed as previously described.^31^, ^32^ Healthy Han Wistar rat's retina (20‐old‐day) was dissected into four equal‐sized pieces, and the explants were separately transferred onto 12‐mm‐diameter filters (0.4 μm pore, Millipore) with the RGC side facing up. The filters were placed into the wells of a 24‐well plate, and each contained 800 μL of culture media. Retinal explant cultures were maintained in humidified incubators at 37°C and 5% CO_2_. Half of the media was refreshed on day 1 and every second day thereafter.

### 2.5 Immunofluorescence staining

Eyes from 6‐month diabetic mice and cultured rat retinal explants were fixed with 4% PFA and snap‐frozen in OCT (VWR Chemicals). For immunostaining, 8‐μm cryosections were washed in PBS and blocked in 5% normal goat serum (NGS), 2% BSA and 0.3% Triton X‐100 in PBS for 30 minutes at RT followed by incubating with the following primary antibodies: mouse anti‐GFAP (#3670, Cell Signaling Technology, 1:200), mouse anti‐Brn‐3a (sc‐8429, Santa Cruz Biotechnology, Inc, 1:50) and rabbit anti‐Rhodopsin (ab3424, Abcam, 1:100)^31^ overnight at 4°C. The secondary antibodies Cy3 conjugated goat antimouse IgG (Merck) and Alexa Fluor 488‐labelled donkey anti‐rabbit IgG (Invitrogen), both with 1:200 dilution in 2% BSA in PBS at RT for 1 hour in the dark. DAPI (Vector Laboratories) was used to show nuclei in sections. Retinal explants were mounted in antifading medium. Images were captured by Leica SP5‐AOBS confocal laser microscope and processed with ImageJ.

For retinal flatmount, the whole retina was permeabilized with 0.2% Triton X‐100 and blocked in PBS with 5% NGS for 2 hours RT. The retinas were then incubated with the primary antibodies (anti‐Brn‐3a [1:50] and anti‐GFAP [1:200]) for 48 hours at 4°C. Tissues were washed before incubation with secondary Alexa Fluor 549‐labelled donkey antimouse IgG (1:200, Invitrogen) for 2 hours at RT, then washed in PBS, flat mounted onto microscope slides and covered with antifading mounting medium (with DAPI) for confocal microscopy.

### 2.6 Quantitative real‐time RT‐PCR

Total RNA extraction was carried out using TRIzol™ Reagent (Life Technologies, UK) according to the manufacturer's instructions. Total RNA was quantified by NanoDrop, and cDNA was synthesized using the ImProm‐IITM Reverse Transcription System (Promega). Then, cDNA was amplified using the Power SYBR^®^ Green PCR Master Mix Reagent (Life Technologies). Primer sequences used were as follows: β‐Actin, forward 5′‐gggaaatcgtgcgtgacattaag, reverse 5′‐tgtgttggcgtacaggtctttg; NPY, forward 5′‐actccgctctgcgacactacat, reverse 5′‐gcgttttctgtgctttccttca; VEGF, forward 5′‐ttactgctgtacctccacc, reverse 5′‐acaggacggcttgaagatg; Angiopoietin‐like 4 protein (ANGPTL‐4) and forward 5′‐ttggtacctgtagccattcc, reverse 5′‐gaggctaagaggctgctgta. The equation fold change = 2−ΔΔct was used for calculation relative changes in expression levels.

### 2.7 TUNEL assay

The terminal deoxynucleotidyl transferase dUTP nick end labelling (TUNEL) assay was performed according to the manufacturer's instructions (Roche). In brief, retinal explants were fixed with 4% PFA and washed in PBS, and retinas were permeabilized with 0.1% Triton X‐100 for 1 hour and rinsed three times with PBS, before incubation with the TUNEL reaction mixture for 1 hour at 37°C. Retinal explants were mounted using antifading mounting medium, and apoptotic cells were observed using Leica SP5‐AOBS confocal laser microscope.

### 2.8 Type 1 diabetic mouse model

All procedures were conducted under the regulation of the United Kingdom Home Office Animals (Scientific Procedures) Act 1986 and were in compliance with the Association for Research in Vision and Ophthalmology statement for the use of animals in ophthalmic and vision research.

Adult (6‐8 weeks) C57BL/6J male mice were obtained from Charles River Laboratories. Type I diabetes was induced by five consecutive days intraperitoneal injections with streptozotocin (STZ) (Sigma‐Aldrich; 40 μg/g bodyweight in 0.1 mol L^−1^ citrate buffer).^33^ Control mice were injected with citrate buffer alone. Diabetes was confirmed by measuring urine glucose (14 days after the onset of diabetic induction, >300 mg/dL considered diabetic; overall disease incidence 88%).

### 2.9 Intravitreal administration of NPY, VEGF and NMDA

In vivo retinal vascular permeability was assessed as previously described.^34^ Briefly, the pupils of 3‐month‐old diabetic mice were dilated using topical 1% tropicamide before induction of anaesthesia. Mice received the following intravitreal injections (2 µL) with a 33‐gauge needle into one eye: VEGF (100 ng); NPY (10 nmol); VEGF with NPY combined; and PBS vehicle. Forty‐eight hours after the first injection, mice received tail vein injections of Evans blue (200 µL, 2%). Mice were killed 10 minutes later, and eyes were fixed with 4% PFA for 2 hours. Retinal flatmounts were mounted in antifading medium, and images were captured by Leica SP5‐AOBS confocal laser microscope and processed with ImageJ.

NMDA (Sigma‐Aldrich) was used to induce excitotoxicity of RGC in the retina,^31^ under experimental conditions described above. A dose‐response was undertaken previously to ascertain the optimal dosing of NPY (5 to 20 nmol); then, mice received the following intravitreal injections (2 µL) with a 33‐gauge needle into one eye: NMDA (10 nmol); NPY (10 nmol); NMDA with NPY together; and PBS vehicle. Mice were killed in 2 days following NMDA injection, and eyes and optic nerves were isolated. Eyes were fixed in 2% PFA for 2 hours for immunofluorescence staining.

### 2.10 Electroretinography (ERG) and optical coherence tomography (OCT) measurements

In vivo imaging was performed in anaesthetized mice using the Micron IV retinal imaging system (Phoenix Research Laboratories). Pupils were dilated with tropicamide (1%) eye drops and positioned on a regulated temperature pad at 37°C to maintain constant body temperature. Subdermal needle electrodes were inserted underneath the skin at the base of the tail (ground electrode) and between the eyes on the forehead (reference electrode). Corneas were lubricated with coupling gel, the objective/electrode was advanced near the corneal surface, and deep red illumination was used to focus on the retina. Responses to focal light stimuli (1mm diameter; spot size D) were recorded at luminance ranging from −0.9 to 3.9 log cd*sec/m^2^. Data are displayed as the mean amplitude (in µV) of the a‐wave (as a measure of photoreceptor function) and b‐wave (as a measure of bipolar cell function).

### 2.11 Statistics

Results are therefore presented as means ± standard deviation (SD). Differences between groups were analysed by Student's *t* test or analysis of variance (ANOVA). All the analysis was performed using GraphPad Prism 6 (GraphPad Software, version 6.01). The significant differences were considered at *P ≤ *.05.

## 3 RESULTS

### 3.1 Diabetes drives neurovascular dysfunction, including Müller cell activation and ganglion cells loss

**Figure 1:**
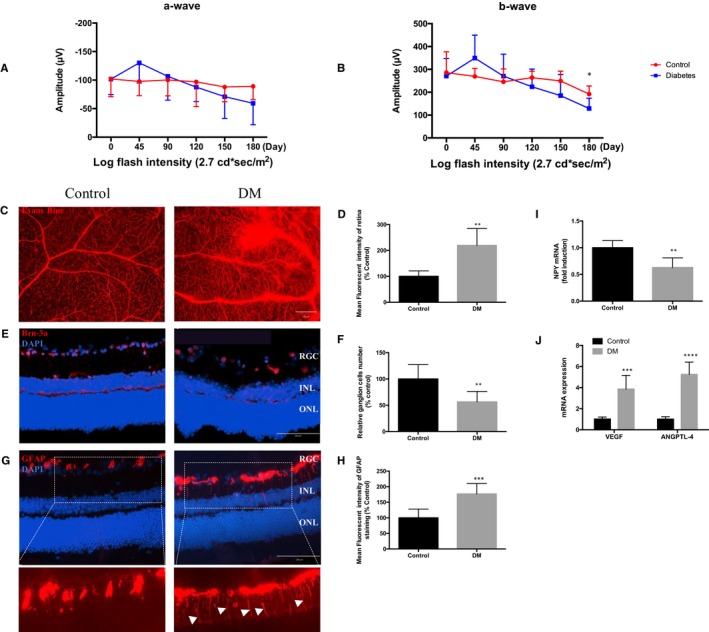
Diabetic retinas exhibit neurovascular dysfunction. Representative images captured from retinal flatmounts and sections obtained from age‐matched wild‐type and 6‐month‐old DM. A, B, ERG recorded a‐wave and b‐wave in scotopic conditions show photoreceptor and inner retina electroretinogram data, respectively. C, Fluorescent images of retina demonstrated differences in vascular integrity (leakage of Evans blue dye, which appears as red). Scale bar = 100 μm. D, The mean fluorescence intensity of Evans blue was quantified using ImageJ and shown to be significantly increased in the DM group. E, Immunostaining of tissue sections retinal ganglion cell marker Brn‐3a (red) counterstained with DAPI (blue). Scale bar, 100 μm. F, Quantification of Brn‐3a+ expression as percentage of control. G, Immunostaining of retinal sections with retinal macroglial cell marker GFAP (red) counterstained with DAPI (blue). Arrows indicate activated Müller cells in diabetic mice. Scale bar, 100 μm. H, The mean fluorescence intensity of GFAP staining was quantified using ImageJ and shown to be significantly increased in the DM group. Retinal ganglion cell layer (GCL), inner nuclear layer (INL) and outer nuclear layer (ONL). I, J, Gene expression in control and diabetic retina demonstrates significant reduction in NPY and increase in pro‐angiogenic factors (VEGF and ANGPTL‐4). Data represent means ± SD of relative values vs control from three independent experiments. *P < .05; **P < .01; ***P < .001; ****P < .0001, statistical analysis was performed with unpaired student's *t* test

To understand how diabetes influences the neurovascular unit in the eye, we first confirmed what alterations to vasculature and neural retina occur in STZ‐induced diabetic model. In vivo monitoring of STZ injected mice and controls permitted longitudinal assessment of retinal function by ERG. There was a steady decline of ERG function in diabetic eyes from 6 to 25 weeks, and by 6 months a significant suppression of the b‐wave, indicative of dysfunctional Müller and bipolar cells (Figure 1A,B). Ex vivo assessment of vascular integrity using retinal flatmount demonstrates leakage of Evans blue dye is significantly increased in the diabetic retina (219.6 ± 29.2%) compared to control retina (100.0 ± 9.4%, *P* ≤ .001; Figure 1C,D). The number of Brn3a + cells, a specific marker of RGC, was quantified on tissue sections and was significantly decreased by 43.6 ± 12.8% of normal controls (Figure 1E,F). In the healthy retina, Müller cells do not express high levels of GFAP, a common marker of reactive gliosis^35^; however, in the diabetic retina an increase MFI of 76.7 ± 17.1% of GFAP expression was observed (Figure 1G,H). Next, we assessed whole retinal tissues for altered expression of gene transcripts encoding NPY and vasoproliferative factors, VEGF and ANGPTL‐4. In 6‐month diabetic retinas, there was significantly reduced NPY mRNA levels detected (Figure 1I), with corresponding increased expression of the pro‐angiogenic VEGF and ANGPTL‐4 (Figure 1J).

### 3.2 NPY regulates the tight junctions of retinal endothelium through MAP kinase

**Figure 2:**
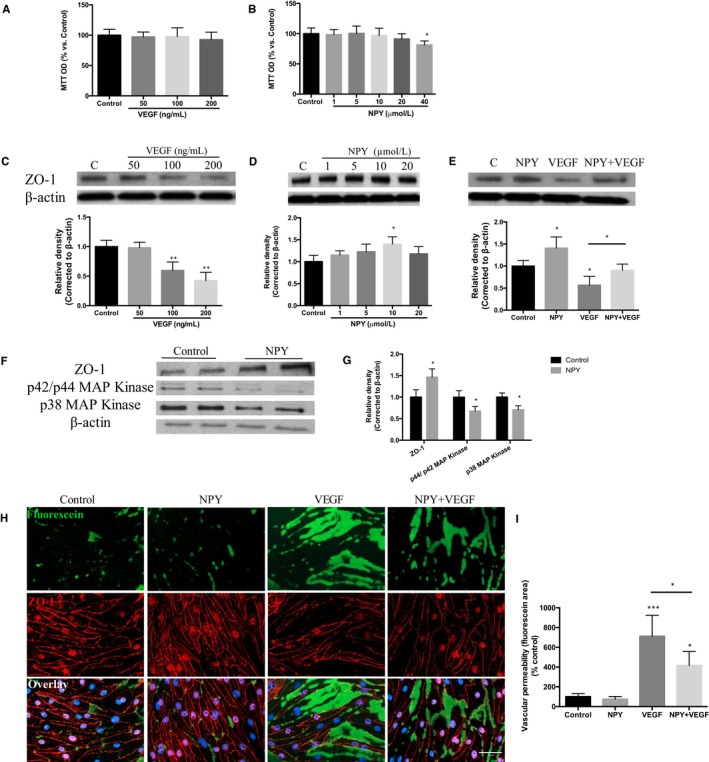
NPY regulates the tight junctions of retinal endothelium through MAP kinase. A, B, MRMEC cell monolayers were serum starved for 24 h, before incubation with complete medium supplemented with NPY or VEGF for a further 24 h. Cell viability was determined in each group using the MTT assay. Data were normalized by control. C, D, SDS‐PAGE analysis demonstrating altered ZO‐1 expression in MRMEC cell lysates treated with NPY (0‐20 μmol L^−1^) or VEGF (0‐200 ng/mL). E, ZO‐1 expression in MRMECs stimulated with NPY (10 μmol L^−1^), VEGF (100 ng/mL) or NPY + VEGF (pre‐incubated with NPY for 6 h before addition of VEGF). F, G, ZO‐1, p44/p42 MAPK and p38 MAPK protein expression in MRMECs treated with NPY (10 μmol L^−1^). H, Immunostaining of MRMECs for fluorescein‐streptavidin to detect permeable areas and for ZO‐1 to show cell‐cell contact; cells deprived of serum for 24 h and incubated in the presence of NPY (10 μmol L^−1^), VEGF (100 ng/mL) or NPY + VEGF (pre‐incubated with NPY for 6 h before addition of VEGF) for 24 h (n = 4 per group). I, Fluorescein intensity associated with DAPI expression was calculated by ImageJ in all treatment groups. Scale bar, 50 µm. Data represent means ± SD of relative values vs control from three independent experiments. *P < .05; **P < .01; ***P < .001; statistical analysis was performed with one‐way ANOVA with Dunn's test for multiple comparisons

The observed reduction in NPY gene expression in the diabetic retina correlates with an increase in vascular permeability and pro‐angiogenic factors. We therefore wished to interrogate the potential role of NPY in maintenance and regulation of the retinal vascular barrier. Using an in vitro approach, we examined the effects of NPY in terms of maintaining the tight junctions in cultured MRMECs. First, we established the response profile of MRMECs following incubation for 24 hours with a range of VEGF and NPY doses (Figure 2A,B). VEGF had no effect on cell viability, and NPY was toxic only at the high dose of 40 µmol L^−1^. SDS‐PAGE analysis of MRMEC cell lysates demonstrated that VEGF induced a dose‐dependent reduction of ZO‐1, whereas NPY increased significantly ZO‐1 expression (Figure 2C,D). For subsequent in vitro assessment, the following doses were used: VEGF 100 ng/mL and NPY 10 µmol L^−1^. At these concentrations, MRMEC expression of ZO‐1 following incubation with VEGF resulted in a significant (43%) reduction, whereas NPY increased expression by 40% compared to untreated controls. When cells were pre‐treated with NPY, applied 6 hours before VEGF, there was a significant increase in ZO‐1 expression (33% higher than VEGF alone) equivalent to expression of the control cells (Figure 2E). To understand the potential mechanism for the protective effect of NPY, we next examined expression of mitogen‐activated protein kinase (MAPK), involving in regulating the expression of tight junction proteins.^36^ Our results indicate that treatment of MRMECs with NPY increased the expression of ZO‐1 and down‐regulated MAPK isoforms p38‐MAPK and p44/p42 MAPK (*P* ≤ .05; Figure 2F,G). NPY was able to reverse the loss of ZO‐1 and increased permeability induced via VEGF.

To demonstrate how NPY‐mediated increase in expression of ZO‐1 influenced the barrier function in MRMECs, we next performed an in vitro vascular permeability assay. This approach permits dual assessment of tight junction expression and extent of monolayer leakage in response to NPY and VEGF treatment. When treating MRMEC cell monolayers with VEGF (100 ng/mL, 24 hours), a 7‐fold increase in fluorescent area (fluorescent‐positive intercellular signal) compared to control (untreated cells) was observed, indicating increased vascular permeability. NPY attenuated vascular permeability through preservation of the integrity of cellular tight junction as demonstrated by a 30% reduction of fluorescent area compared to VEGF alone (*P* < .05) (Figure 2H,I). In addition, ZO‐1 immunostaining confirmed the breakdown integrity of tight junction following VEGF treatment, which was similarly partially prevented by NPY pre‐treatment (Figure 2H). To assess whether NPY modulation of murine endothelial cells could be extended to human and thus bring a translational understanding of the role of NPY, experiments were also performed using HMRECs, demonstrated similar protective effects and maintained ZO‐1 expression (Figure S1A). To model microvascular disturbance observed in the hyperglycaemic retina,^37^ we determined the angiogenic potential of HRMECs in response to NPY under high glucose (HG) conditions. Utilizing the in vitro tube formation assay, quantitative analysis showed that HRMEC tube length was significantly reduced with HG exposure (50 mmol L^−1^) compared with control group (5.5 mmol L^−1^ glucose), whilst NPY co‐treatment under HG conditions maintained the angiogenic capacity (*P* < .05; Figure 1B,C).

### 3.3 NPY inhibits VEGF‐induced vascular permeability in diabetic mice

**Figure 3:**
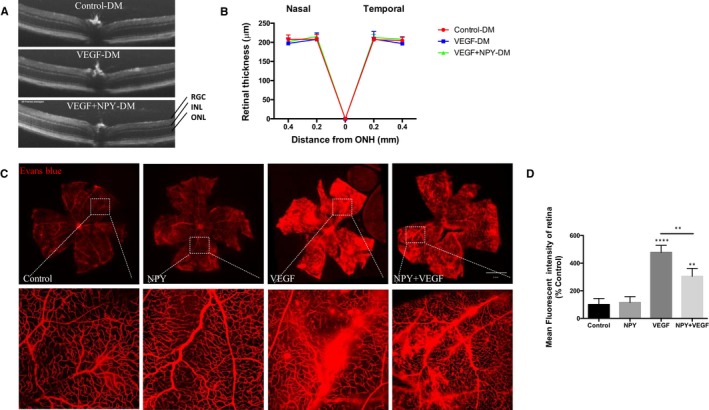
NPY inhibits VEGF‐induced vascular permeability in diabetic mice. Groups of 3‐month‐old diabetic mice received intravitreal injections of NPY (10 nmol), VEGF (10 nmol) or NPY in combination with VEGF (n = 6 per group). A, B, At 24 h post‐injection, clinical OCT images were acquired, and retinal thickness quantified. Data are representative of two measurements per retina. C, Representative ex vivo retinal flatmount images prepared 48 h following treatment demonstrate differences in the vasculature (leakage of Evans blue dye, which appears as red). Scale bar = 1 mm. D, The fluorescent intensity of Evans blue was quantified by ImageJ. **P < .01; ****P < .0001, statistical analysis was performed with one‐way ANOVA with Dunn's test for multiple comparisons

The in vitro data demonstrate that NPY can modulate VEGF‐induced vascular changes, so we next investigated whether this protective effect on vascular integrity translated to an in vivo setting. Groups of 6‐month‐aged diabetic mice were treated with a single intravitreal injection of VEGF (100 ng), NPY (10 nmol) or NPY + VEGF and clinically assessed at 48 hours post‐injection. Clinical OCT assessment demonstrated that VEGF or the combined VEGF + NPY treatments did not alter structural integrity as measured by retinal thickness (Figure 3A,B).

Vascular leakage was then assessed by extravasation of intravascular Evans blue dye (EB) in the different treatments. Retinal flatmounts exhibited that intravitreal injection of VEGF results in a 4.8‐fold increase in extravasation of Evans blue as confirmed via mean fluorescent intensity compared to control. Intravitreal injection of NPY together with VEGF decreased the leakage in retinal vessels by 36% compared to VEGF alone (Figure 3C,D). On the contrary, minimal vascular permeability of EB was observed in retinas receiving vehicle (PBS control) or NPY alone injections (*P* < .01).

### 3.4 NPY protects retinal ganglion cells against apoptotic cell death induced by NMDA ex vivo and in vivo

As NPY receptors are expressed in the inner retina and recognition that RGC are perturbed in diabetes,^38^ we then wished to evaluate whether NPY played a homeostatic role and exerted a protective effect to preserve RGC. To investigate this further, the NMDA‐induced RGC excitotoxic model was employed in retinal explants.^31^

**Figure 4:**
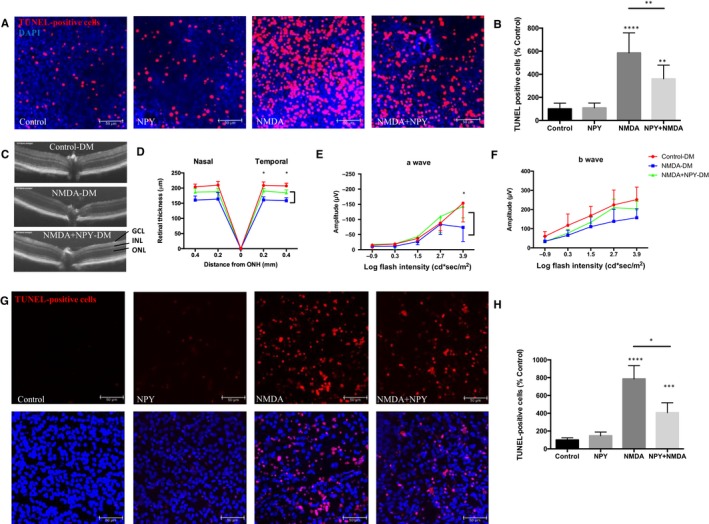
NPY protects retinal ganglion cells against apoptotic cell death induced by NMDA. A, B, Representative images showing TUNEL + cells in rat retinal explants exposed to NMDA (10 µmol L^−1^), NPY (10 µmol L^−1^) or pre‐treated with NPY 6 h before NMDA exposure, showing TUNEL+ (red) and cell nuclei stained with DAPI (blue). Scale bar, 50 µm. Quantification of TUNEL + cells expressed as percentage of control. Groups of 3‐month‐old diabetic mice received intravitreal injections of NPY (10 nmol), NMDA (10 nmol) and NPY in combination with NMDA (n = 6 per group). C, D, At 48 h post‐injection, OCT images show intravitreal NMDA results in reduced retinal thickness and NPY partially protects. ONH = optic nerve head. Data are representative of two measurements per retina. E, F, ERG a‐wave and b‐wave responses represented by mean values of amplitudes in scotopic conditions. G, H, Representative images of retinal whole mounts prepared for TUNEL staining and ImageJ analysis of confocal images. *P < .05; **P < .01; ***P < .001; ****P < .0001, statistical analysis was performed with one‐way ANOVA with Dunn's test for multiple comparison, and EGF was analysed using two‐way ANOVA for multiple comparison

TUNEL + cells were quantified indicate NMDA treatment leads to a 4.8‐fold increase in apoptotic cell number. Treating explants for 6 hours with NPY (10 µmol L^−1^) prior to exposure to NMDA decreased the cytotoxic effects, as presented by a significant reduction in TUNEL + cells by 38% as compared to NMDA alone (*P* < .05; Figure 4A, B). NPY treatment alone results in comparable low TUNEL + cells in comparison with control retinal explants.

To further elucidate with an in vivo correlate, we analysed flatmounts of diabetic eyes exposed to NMDA for 24 hours as a model of induced neuronal excitotoxic damage. A dose‐response was tested prior to confirm the optimal dosing of NPY (5 to 20 nmol), which showed that a 10 nmol NPY dose provided the optimal protective effect in perturbing NMDA‐induced RGC apoptosis model (Figure S2A,B). In diabetic eyes, administration of NMDA alone reduced the overall total retinal thickness by 23% compared to disease controls. However, the combined administration of NPY and NMDA the extent of cell loss is significantly reduced by 16% compared to NMDA alone (*P* < .05; Figure 4C,D). Similarly, ERG responses demonstrate that eyes receiving a combined NPY and NMDA injection have improved functional responses, with increased a‐wave and b‐wave, compared to NMDA alone, although these were not statistically significant in b‐wave (Figure 4E,F). Retinal wholemount assessment from diabetic eyes receiving NMDA shows a 7.9‐fold increase in TUNEL + cells compared to control. The combined NPY injection leads to a significant reduction in TUNEL + cells (from 7.9‐fold to 4.1‐fold of control; *P* < .05; Figure 4G,H).

### 3.5 NPY restored neurovascular function in diabetic retinas in vivo

**Figure 5:**
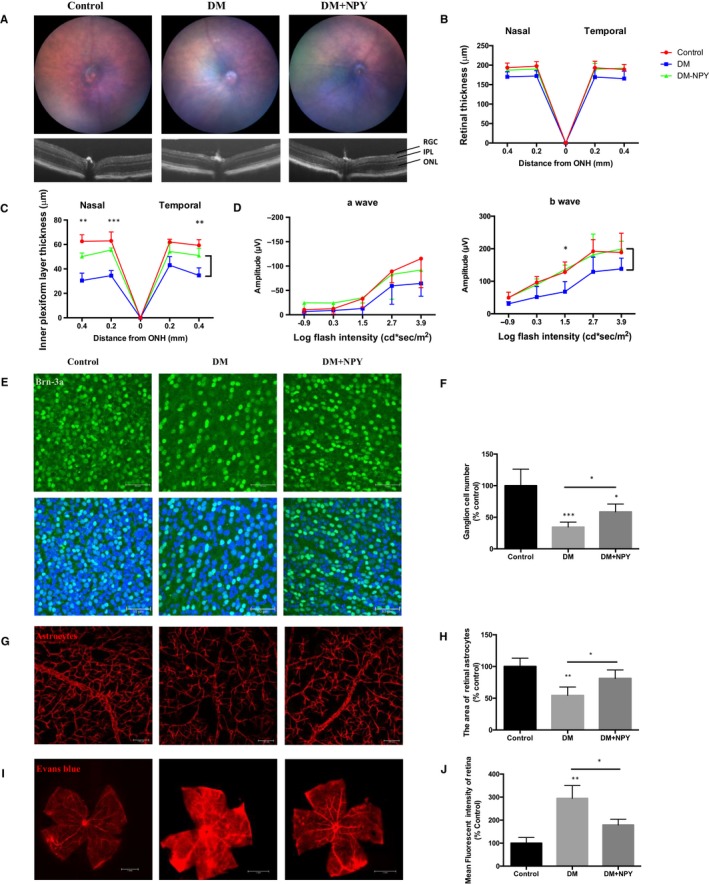
NPY restores neurovascular function in diabetic retinas. Groups of diabetic mice received intravitreal injections (NPY; 10 nmol) at 3 and 5 mo. A‐C, Representative fundal and OCT images captured at 6 mo, showing no difference in retinal thickness between NPY‐injected eyes and age‐matched control eyes. The total retinal thickness is significantly reduced in the diabetic retina, primarily in IPL. D, ERG a‐wave and b‐wave responses represented by mean values of amplitudes in scotopic conditions. E, F, Representative confocal images of retinal whole mounts and quantification of Brn‐3a immunostaining, showing that NPY prevents the loss of retinal ganglion cells in diabetic mice. G, H, Representative images and quantification of GFAP immunostaining, showing that NPY also protects against the loss of retinal astrocytes. I, J, Representative images 48 h following treatment demonstrate differences in the vasculature (leakage of Evans blue dye, which appears as red). Scale bar = 50 μm. The fluorescent intensity of Evans blue was quantified by ImageJ. *P < .05; **P < .01; ***P < .001; statistical analysis was performed with one‐way ANOVA with Dunn's test for multiple comparisons, and EGF was analysed using two‐way ANOVA for multiple comparison

To determine whether the positive effects of NPY extended to offer long‐term protection in the diabetic retina, NPY was administered via intravitreal injection at 3 and 5 months post‐induction of diabetes. Clinical assessment by OCT to measure retinal thickness and ERG to determine changes in retinal function was performed at regular intervals throughout the experimental time course. Representative clinical assessment at 4 months (Figure S3) showed no significant changes to the fundal appearance, total retinal thickness or ERG response between NPY‐injected eyes and age‐matched control eyes. Whilst the total retinal thickness was slightly reduced in the diabetic eyes, this was not significant. At 6 months following induction of diabetes, there was no difference in retinal thickness in the NPY‐injected eyes compared to age‐matched control eyes. However, the inner plexiform layer thickness in DM groups was reduced by 42% compared to control group and the inner plexiform layer thickness in the NPY treated group was increased by 47% compared to DM group. Cell loss occurred predominantly in the inner plexiform layer (IPL; Figure 5A‐C). ERG assessment also demonstrates that NPY administration increased a‐wave and b‐wave amplitudes to levels equivalent to age‐matched non‐diabetic controls (*P* < .05; Figure 5D). These data support a notion that diabetic eyes injected with NPY had sustained neuronal cell function. To confirm whether the protective effect of NPY was evident in ganglion cells and astrocytes, retinal flatmounts were prepared for ex vivo immunohistochemistry assessment. Quantification of Brn‐3a + RGC numbers in the diabetic retina shows a 66% reduction in cell numbers compared to age‐matched controls. By contrast, DM eyes treated with NPY demonstrate a significant increase in Brn‐3a + cells (from 34% to 59% of control) (*P* < .05; Figure 5E,F). Retinal glial coverage was evaluated by staining with GFAP, showing widespread loss of glial coverage was observed in diabetic retina (Figure 5G). The relative glial coverage in the diabetic retina was significantly reduced by 55% compared to control retina. The diabetic eyes treated with NPY demonstrated a significant increase by 49% in glial cell coverage compared to untreated diabetic eyes (*P* < .05; Figure 5H). Vascular integrity assessment by extravasation of EB dye showed a 3‐fold increase in the diabetic retina as compared to control eyes, and this was remarkably reduced in the NPY‐treated group (*P* < .05; Figure 5I,J).

## 4 DISCUSSION

In this study, we demonstrate a protective role of NPY in the retina, preventing loss of RGC and vascular leakage in diabetic mice in vivo. These findings are supported by in vitro and ex vivo evidence of NPY‐mediated protection of MRMECs and RGC. The data support how modulation of neurovascular unit may offer therapeutic opportunities to prevent progression of DR.

The pathogenesis of DR is highly complex and a multifactorial process. Hyperglycaemia perturbs the metabolic and haemodynamic equilibrium, altering the molecular signature of multiple different cell types, principally endothelial cells and pericytes in the retina. Diabetes elicits the formation of advanced glycation end products and reactive intermediates of altered metabolism, increased oxidative stress, which ultimately drives progressive neural and vascular damage.^39^, ^40^, ^41^ There are also reported data suggesting that DR results from changes to the neurovascular unit rather than isolated neuroglial or vascular alterations.^42^, ^43^ The in vivo clinical assessment detailing the changes in ERG response and OCT retinal thickness in the DM mice supports previous reports which demonstrate a progressive neuronal injury at 6 weeks after induction of diabetes.^44^ Whilst the ERG technology available permitted reproducible and reliable recording of a‐wave and b‐wave, to fully evidence the contribution of RGCs precise measurements of photopic negative responses are required. As ERG reflects the overall function of the retina (dependent on intact and functioning RGC), we assessed Brn‐3a expression and the change in RGC number as an indirect surrogate of function. Furthermore, in addition to vascular disruption we have shown there is significant neuronal impairment (reduced numbers of Brn‐3a + RGC and activation of GFAP + retinal glial) at 6 months, which supports that dysfunction of retinal neurons occurs early following the onset of diabetes. Considering the current data together with previous reports, there is compelling evidence to support that DR is a consequence of neurovascular unit disruption. Reduction of NPY expression (as we demonstrate in DM mice) may be associated with increased retinal neuron apoptosis and development of DR. This would suggest that normal levels of NPY are critical to the homeostasis and maintenance of neurovascular unit.

Increased vascular permeability in diabetes is recognized as a complex process involving multiple signalling pathways, mediated principally by VEGF which disrupts the integrity of endothelial cell‐cell junctions.^45^ Among the various protein components of tight junctions, ZO‐1 is a phosphoprotein that participates in multiple protein‐protein interactions regulating tight junction integrity.^46^ VEGF disrupts tight junctions by altering phosphorylation of ZO‐1 and occluding through a Src‐dependent pathway.^47^ In our experiments, and in accordance with previous work,^48^ we demonstrate the disruptive effects of VEGF on the expression of tight junction protein ZO‐1 in endothelial cells. Moreover, we demonstrate that VEGF and NPY have opposite effects modulating the expression of ZO‐1 in vitro using the MRMEC cell monolayers. Treatment with NPY increased expression of ZO‐1, accompanied by reduction of the phosphorylated MAPK isoforms, supporting our initial hypothesis that NPY maintains vascular integrity, and this is dependent on MAPK inhibition. The PKC‐MAPK‐NF‐κB pathway is recognized to modulate the expression of tight junction proteins and the barrier function in various organs or cells.^49^ Targeted inhibition of protein kinase C zeta (PKCζ) reduces NF‐κB activation and protects expression tight junction complex and cell permeability in retinal endothelial cells.^50^ In addition, NPY has been shown to inhibit nuclear translocation of NF‐κB in microglia challenged with IL‐1β, thus preventing IL‐1β‐induced nitric oxide release and a further mechanism to protect and regulate the endothelial barrier function.^51^, ^52^

Our previous studies using in vitro vascular permeability assay clearly demonstrate VEGF mediated increase in retinal endothelial cell monolayer permeability,^31^ and when the assay was deployed here, results show that NPY suppressed VEGF‐induced monolayer leakage and permeability. In addition, NPY treatment enhances stability of ZO‐1 proteins at cell‐cell contacts in human retinal endothelial monolayer, coincident with leakage of fluorescence. Our data suggest the protective effect of NPY on the integrity of the retinal endothelial barrier is likely due to its ability to induce stabilization of tight junction complexes. Furthermore, and relevant to DR, our observations from the diabetic mice demonstrate the protective role of NPY against VEGF‐induced microvascular permeability in vivo. The protective effects of NPY are similar to another neuropeptide, substance P,^53^ which we previously demonstrated can maintain and support retinal vascular integrity.

Other alterations of diabetic retina include the increased frequency of apoptosis of retinal cells. Barber and co‐workers^38^ previously documented increased apoptosis of retinal ganglion cells (RGC) in experimental diabetes in rats and diabetes mellitus in humans. Similarly, we report that in the retinas of STZ‐induced DM, there is a significant decrease of retinal thickness in DM compared with controls. Interestingly, segmentation analysis of retinal OCT images indicates cell loss occurs primarily in the retinal inner plexiform layer (IPL), a layer comprising a dense reticulum of fibrils predominantly formed by interlaced dendrites of RGC. There is evidence that ganglion cell death in DR occurs via glutamate‐mediated toxicity.^54^ Glutamate is the major excitatory neurotransmitter in the retina and is involved in neurotransmission from photoreceptors to bipolar cells and from bipolar cells to ganglion cells. Elevated levels of glutamate are implicated in neurodegeneration and its toxic effects on the retina, particularly in RGC are well‐established.^55^, ^56^ In both human diabetes and experimental models of diabetes, glutamate levels are shown to be elevated.^54^, ^57^, ^58^ Expression of NPY in the mouse retina affords neuroprotective effects,^26^ confirmed by in vitro observations that rat retinal cells are protected from NMDA‐induced toxicity,^59^ although the NPY receptor subtype(s) involved in this neuroprotective effect remain unknown. In the current study, we extend understanding of the beneficial effects and show that NPY exerts neuroprotective properties on RGC in NMDA‐induced neuro‐excitotoxicity apoptotic cell death both in ex vivo retinal explants and in the diabetic animal model. Whilst arguably switching of species may confound, we selected to use the rat retinal explant model as an established, more easily manipulated and robust assay for RGC assessment.^31^, ^60^, ^61^ Although the RGC subtypes vary between species, the anatomical location and number are largely equivalent.

In terms of the intracellular mechanisms underlying the NPY neuroprotective role against cell death induced by glutamate, the protective effects of NPY are thought to be linked to inhibition of glutamate release, as reported in the CNS, specifically the hippocampus.^23^, ^25^ In rat retinal cultures, NPY inhibits both the [Ca^2+^] increase induced by KCl^62^ and the aspartate release in these cultures (unpublished observations).^26^ Different intracellular signalling pathways are reported to facilitate NPY‐mediated neuroprotection and the involvement of ERK1/2 and Akt pathways in Parkinson's disease model,^63^ as well as PKA and p38K in retinal neural cells.^26^ In terms of neuroprotection, we speculate that NPY similarly exerts a protective effect through MAPK signalling, as MAPK has been shown to regulate tight junction function. Future work in our laboratory will explore those questions.

In contrast, recent publications suggest NPY does not exert a protective effect in the retina^29^ and that NPY shows no association at gene level (SNP in NPY2R gene) in patients with diabetes.^64^ These reports utilize notably different models, addressing different questions but taken together, may suggest that NPY signalling is context‐dependent, with the potential to exert both beneficial neuroprotective and negative effects. The study by Christiansen demonstrates that NPY has a detrimental effect in ischaemic retina, a model induced by acutely raising intraocular pressure and restricting ocular perfusion. However, in diabetic retinas (and seen in the STZ model) more insidious pathophysiological changes are observed, which show reduced NPY (gene and protein) expression in the mouse retina,^30^ more relevant to human diabetes, and emphasize the importance of the context and how the results are interpreted. In the current study, our approach was to investigate the adjunctive effect of extracellular NPY as a therapy to restore homeostasis, opposed to an association with a specific SNP in the NPY gene that may indicate causality in DR. The lack of genetic association reported in diabetic patients does not prevent therapeutic exploitation of the NPY pathway.

Taken together, these studies highlight the regulatory and homeostatic role of the neurovascular unit that is disrupted in DR. The current data support that adjunctive NPY therapy sustains neuronal health and can attenuate at least in models the downstream effects of diabetic retinal vasculopathy, notably vascular leakage and neuronal death, as well as the loss of Glia cells.

## CONFLICT OF INTEREST

The authors declare that there is no conflict of interests regarding the publication of this paper.

## AUTHORS' CONTRIBUTIONS

Kepeng Ou, Dave A Copland, Lei Liu and Andrew D Dick designed this study. Kepeng Ou, Dave Copland and Youjian Li carried out experiments. Dave A Copland, Sofia Theodoropoulou and Jian Liu provided experimental methods. Kepeng Ou took on the statistical analysis. Kepeng Ou, David A Copland, Sofia Theodoropoulou, Sonja Mertsch, Stefan Schrader, Lei Liu and Andrew D Dick drafted the manuscript. David A Copland, Sofia Theodoropoulou, Lei Liu and Andrew D Dick provided critical suggestions and revised the manuscript. All the authors had the approval of the submitted and published versions.

## Data Availability

The data used to support findings of the study are available from the corresponding author upon request.
