# Supplementary figures and images for: Treatment of diabetic retinopathy through neuropeptide Y‐mediated enhancement of neurovascular microenvironment
Source: J Cell Mol Med. 2020 Mar 6;24(7):3958–70. doi: 10.1111/jcmm.15016 (PMC7171318; doi:10.1111/jcmm.15016)

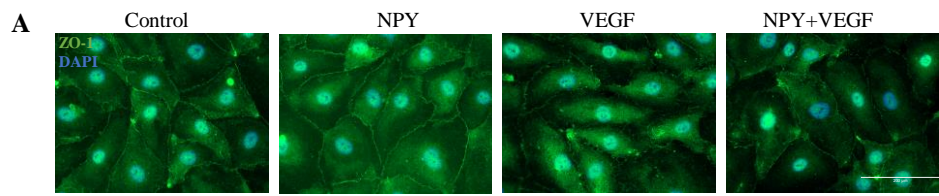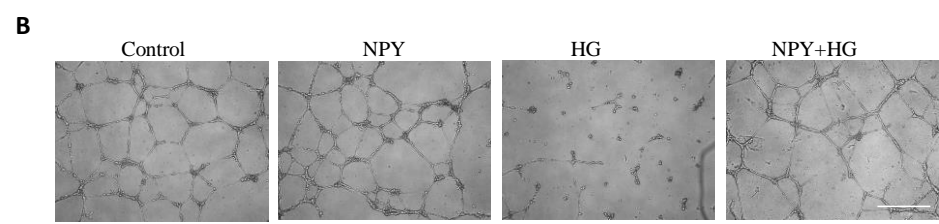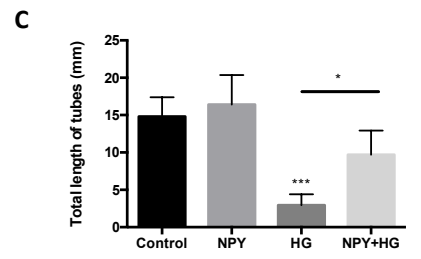

Supplementary 1

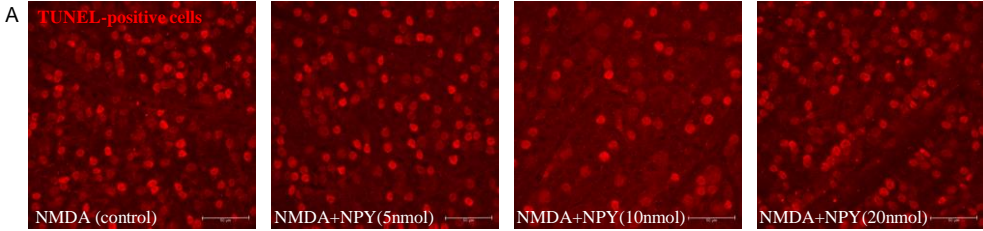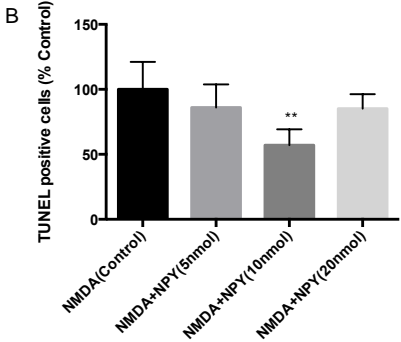

Supplementary 2

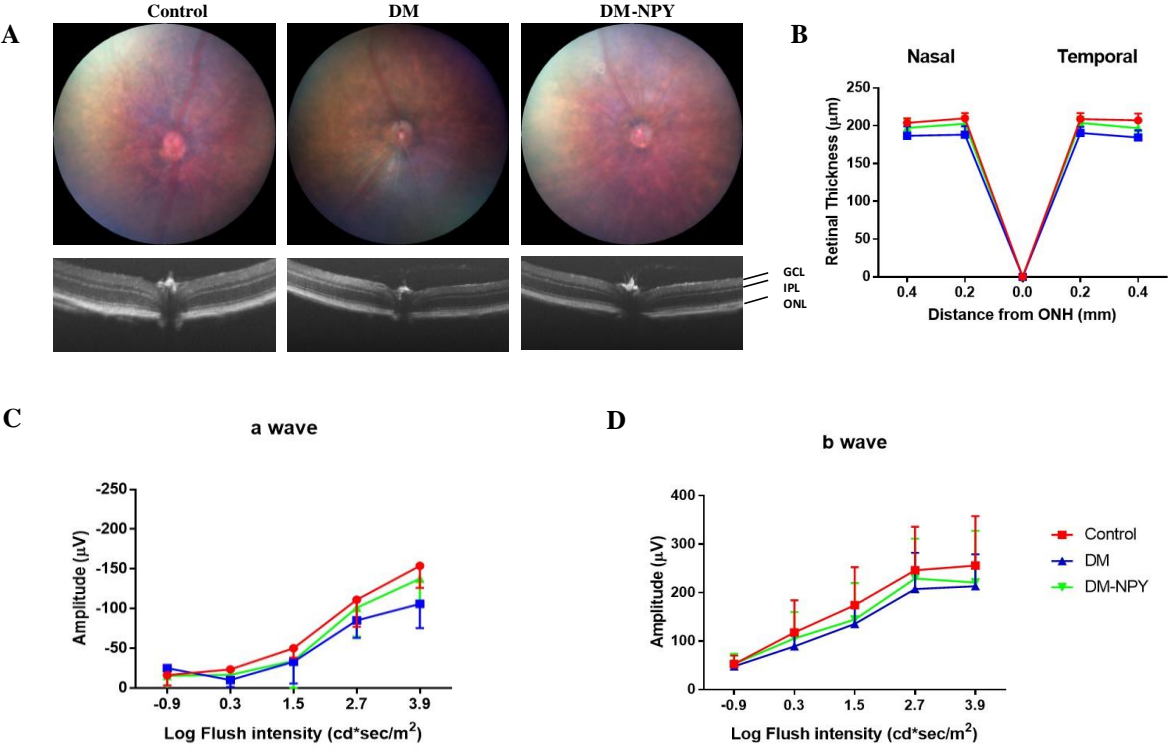

Supplementary 3

Supplement: Supplementary file 1 [file JCMM-24-3958-s001.pdf]
