# Supplementary material for: Treatment of diabetic retinopathy through neuropeptide Y‐mediated enhancement of neurovascular microenvironment
Source: J Cell Mol Med. 2020 Mar 6;24(7):3958–70. doi: 10.1111/jcmm.15016 (PMC7171318; doi:10.1111/jcmm.15016)
Supplement: Supplementary file 2 [file JCMM-24-3958-s002.docx]

**Supplementary Fig. 1.** (**a**) NPY stabilizes ZO-1 expression *in vitro* following VEGF-induced permeability. Representative pictures of confluent HRMEC cultures at 24 hours following treatment with NPY (10 µM), VEGF (100 ng/ml) alone or NPY followed by VEGF 6 hours later stained for ZO-1 (green) and nuclei (DAPI). Scale bar 200 µm. (**b, c**) Representative bright-field images showing HRMEC tube formation. Addition of HG (50 mM) alone inhibited tube formation, whereas the addition of NPY (10 µM) in combination with HG only partially inhibits. Scale bar, 500 µm. Data represents means ± SD of relative values vs control from 3 independent experiments. **P* < 0.05; *** *P* < 0.001; statistical analysis was performed with one-way ANOVA with Dunn’s test for multiple comparisons.

**Supplementary Fig. 2.** (**a**) Representative images of retinal whole-mounts prepared for TUNEL assay, 24 hours following a single intravitreal injection of NMDA (10 nmol), different dose of NPY (5 nmol, 10 nmol and 20 nmol) combined with NMDA (n=4 per group). (**b**) TUNEL+ was quantified by Image J, showing that NPY (10 nmol) significantly decreases the intensity of apoptotic retinal ganglion cells induced by NMDA. There was no significant difference between the two concentrations (10 nmol and 20 nmol) to demonstrate whether there was a dose response. Scale bar, 1mm. ***P* < 0.01, statistical analysis was performed with one-way ANOVA with Dunn’s test for multiple comparisons

**Supplementary Fig. 3.** Groups of diabetic mice received intravitreal injections (NPY; 10 nmol) at 3-months. (**a, b**) Representative fundal and OCT images captured at 4-months, showing no difference in retinal thickness between NPY-injected eyes and age-matched control eyes. The total retinal thickness is slightly reduced in the diabetic retina. (**c, d**) ERG a- and b-wave responses represented by mean values of amplitudes in scotopic conditions, EGF were analysed using two‐way ANOVA for multiple comparison.
